# Melanoma incidence, recurrence, and mortality in an integrated healthcare system: A retrospective cohort study

**DOI:** 10.1002/cam4.2252

**Published:** 2019-06-19

**Authors:** Heather S. Feigelson, John D. Powers, Mayanka Kumar, Nikki M. Carroll, Arun Pathy, Debra P. Ritzwoller

**Affiliations:** ^1^ Institute for Health Research, Kaiser Permanente Colorado Aurora Colorado; ^2^ Department of Dermatology Kaiser Permanente Colorado Aurora Colorado

**Keywords:** Incidence, melanoma, mortality, prognosis, recurrence

## Abstract

**Background:**

Numerous studies have examined melanoma incidence and survival, but studies on melanoma recurrence are limited. We examined melanoma incidence, recurrence, and mortality among members of Kaiser Permanente Colorado (KPCO) between January 1, 2000 and December 31, 2015.

**Methods:**

Age‐adjusted incidence rates were computed to examine trends among KPCO members aged 21 years and older. Cox proportional hazards models were used to examine factors associated with recurrence and mortality.

**Results:**

Our cohort included 1931 cases of invasive melanoma. Incidence rates increased over time and were higher than SEER rates; however, the increase was limited to early stage disease. In multivariable models, stage at initial diagnosis, gender, and age were associated with melanoma recurrence. Men were more likely to have a recurrence than women (adjusted hazard ratio [HR]: 1.70, 95% confidence interval [CI]: 1.19‐2.43), and for each decade of increasing age, the adjusted HR = 1.20 (95% CI: 1.06‐1.37). Factors associated with all‐cause mortality included stage (HR = 12.87, 95% CI: 6.63‐24.99, for stage IV vs stage I), male gender (HR = 1.42, 95% CI: 1.12‐1.79), older age at diagnosis, lower socioeconomic status, and comorbidity index. For melanoma‐specific mortality, results were similar, with one exception: age was not associated with melanoma‐specific death (HR = 1.09, 95% CI: 0.94‐1.25, *P* = 0.253).

**Conclusions:**

Data derived from an insured patient population, such as KPCO, have the potential to enhance our understanding of emerging trends in melanoma. This is the first population‐based study in the United States to examine patient characteristics associated with risk of recurrence. Men have an increased risk of both recurrence and death, and thus may benefit from more intensive follow‐up than women.

## 1 BACKGROUND

In the United States, cutaneous melanoma (hereafter referred to as melanoma) is the fifth most common cancer in men and the sixth most common cancer in women, with an estimated 91 270 new cases in 2018.^1^ Over the past 30 years, the incidence of melanoma has risen rapidly, although current trends suggest this pattern may be shifting in certain age groups.^2^, ^3^ A recent report using data from National Cancer Institute's Surveillance, Epidemiology, and End Results (SEER) Program through 2014 found increasing incidence rates in older men and women, but decreasing rates in younger age groups.^2^ Death from melanoma also appears to be on the decline, especially among younger patients.^1^ The 5‐year relative survival rate for melanoma is 91%, and even higher (98%) among those diagnosed with localized disease. Five‐year survival rates for individuals with regional and distant‐stage diseases are 62% and 16%, respectively.^4^

Despite these encouraging trends related to improvements in screening and the introduction of novel therapeutics,^5^, ^6^ melanoma remains a significant public health problem, with an estimated 1.2 million melanoma survivors currently living in the United States.^4^ While many previous studies have examined factors associated with survival,^7^, ^8^, ^9^, ^10^, ^11^, ^12^, ^13^, ^14^, ^15^, ^16^, ^17^ little is known about factors associated with risk of recurrence.^18^, ^19^, ^20^, ^21^, ^22^, ^23^ Data on cancer recurrence can yield important information that can guide treatment and surveillance planning, but is often unavailable. The Kaiser Permanente Colorado (KPCO) tumor registry tracks cancer cases for recurrence,^24^, ^25^ as well as for mortality, providing a unique opportunity to examine characteristics associated with melanoma recurrence. Data derived from an insured, monitored patient population, such as KPCO, have the potential to enhance our understanding of emerging trends in melanoma, including differences by age and gender. In this retrospective cohort, we examine incidence, recurrence, and mortality among KPCO members diagnosed with melanoma from January 1, 2000 through December 31, 2015.

## 2 METHODS

Kaiser Permanente Colorado has provided comprehensive health care to the greater Denver metropolitan area since 1969 and currently provides care to approximately 12% of the state's entire population (over 600 000 people). The primary data source for this analysis was KPCO's tumor registry that is linked to a variety of electronic health record (EHR) records and other administrative data. These data have been extracted and loaded into relational tables, known as the “virtual data warehouse” and linked through a common, unique identifier.^26^, ^27^, ^28^, ^29^

The tumor registry contains data consistent with the North American Association of Central Cancer Registries (NAACCR) standard. Tumor registry data, including first course treatment, were obtained from manual reviews of patients’ medical charts by certified tumor registrars and include coded clinical data associated with inpatient and outpatient events. Stage at diagnosis was determined using the American Joint Committee on Cancer (AJCC) Cancer Staging Guidelines. A unique feature of the KPCO tumor registry is its tracking of cancer recurrence. Patient charts are manually reviewed specifically to access recurrence status. A recurrence of melanoma is defined as a detected melanoma that occurs after a patient is declared disease free after completion of definitive therapy (eg, excision, radiation, and/or chemotherapy). For this analysis, all melanoma cases had manual chart review by a certified tumor registrar at the start of the study to verify recurrence status.

Patient characteristics not available in the tumor registry were extracted from the virtual data warehouse. As an indicator of general health, we used the Quan adaptation of the Charlson Comorbidity Index^30^ derived from diagnosis codes captured from all inpatient claims and EHR and encounters that occurred 12 months prior to melanoma diagnosis. Data on cause and date of death were derived from the tumor registry, membership data, state‐level mortality files, and Social Security Administration data. We used the patient's home address mapped to census block level information on education to estimate socioeconomic status. Socioeconomic status was expressed as the percent of people in the census block with a college (or higher) education.

This project was approved by the KPCO Institutional Review Board. All analyses were performed by SAS 9.2 and 9.4 (SAS Institute, Cary, NC).

### 2.1 Statistical analysis

Patients diagnosed with stage I‐IV melanoma between January 1, 2000 and December 31, 2015 were identified from the tumor registry and followed through December 31, 2017 for recurrence and mortality. We excluded in situ cases, those who were less than 21 years of age, who were missing information on race/ethnicity or stage, or who were not enrolled at KPCO at the time of diagnosis.

Patient characteristics were reported as means or medians with standard deviations for interval‐level variables, and percentages for categorical variables. Age‐adjusted incidence was calculated using the age distribution of the US 2000 population as the standard. We compared our incidence rates to SEER (version 18) data (www.seer.cancer.gov).

Cox proportional hazards models were used to estimate both risk of recurrence and death while adjusting for important covariates. Analysis of recurrence was limited to patients diagnosed with stage I‐III at diagnosis and declared disease free after first course therapy. Time to recurrence was defined as months from diagnosis until recurrence. Both all‐cause and melanoma‐specific mortality were examined; survival time was defined as months from diagnosis until death. Patients who disenrolled from the health plan, had a subsequent cancer, or who were alive at the end of the follow‐up period (December 31, 2017) were censored on those dates.

The following covariates were included in the survival analysis: stage at diagnosis, gender, age (as a continuous variable), race/ethnicity (white or non‐white), socioeconomic status as measured by percent of college educated individuals in census tract of residence, comorbidity scores (0, 1‐2, or ≥3 comorbid conditions), and treatment. Treatment was classified into four dichotomous variables based on data in the tumor registry: receipt of surgery (yes/no), radiation therapy (yes/no), chemotherapy (yes/no), or biologic response modulators (yes/no). Treatment categories were not mutually exclusive.

Variables included in the analysis of recurrence were the same as those in the survival analysis, with the exception of surgery because all but one case in the recurrence analysis had surgery as part of their treatment. To examine the effects of treatment on recurrence, we constructed multivariable models with and without treatment.

Finally, we conducted a sensitivity analysis for both recurrence and all‐cause mortality using tumor thickness rather than disease stage among the sub‐set of cases where thickness data was available.

## 3 RESULTS

A total of 1,931 KPCO members 21 years of age or older were diagnosed with invasive melanoma from 2000 to 2015. Table 1 shows the demographic and tumor characteristics of the study population overall and by stage. Most cases were diagnosed at stage I (77.5%). More cases occurred in men (57.8%) than in women (42.2%), and our cohort was predominantly non‐Hispanic white (97.8%). Most patients (97.7%) were treated with surgery; use of other treatment modalities was uncommon. However, among cases diagnosed at stage IV (N = 54), radiation, chemotherapy, and biologic response modulators were each used in about a quarter of patients.

**Table 1:** Characteristics of invasive melanoma cases, Kaiser Permanente Colorado, 2000‐2015

Characteristic	Total cases	Stage I	Stage II	Stage III	Stage IV
	N (%)	N (%)	N (%)	N (%)	N (%)
Number of people	1931	1497 (77.5%)	277 (14.3%)	103 (5.3%)	54 (2.8%)
Age group					
<30	57 (3.0%)	45 (3.0%)	7 (2.5%)	5 (4.9%)	0 (0.0%)
30‐39	148 (7.7%)	124 (8.3%)	13 (4.7%)	6 (5.8%)	5 (9.3%)
40‐49	250 (13.0%)	209 (14.0%)	25 (9.0%)	11 (10.7%)	5 (9.3%)
50‐59	398 (20.6%)	333 (22.2%)	33 (11.9%)	22 (21.4%)	10 (18.5%)
60‐69	483 (25.0%)	375 (25.1%)	59 (21.3%)	31 (30.1%)	18 (33.3%)
70‐79	387 (20.0%)	269 (18.0%)	85 (30.7%)	22 (21.4%)	11 (20.4%)
80+	208 (10.8%)	142 (9.5%)	55 (19.9%)	6 (5.8%)	5 (9.3%)
Gender					
Female	815 (42.2%)	665 (44.4%)	97 (35.0%)	34 (33.0%)	19 (35.2%)
Male	1116 (57.8%)	832 (55.6%)	180 (65.0%)	69 (67.0%)	35 (64.8%)
Race/Ethnicity					
Non‐Hispanic White	1888 (97.8%)	1466 (97.9%)	269 (97.1%)	102 (99.0%)	51 (94.4%)
Hispanic	21 (1.1%)	13 (0.9%)	5 (1.8%)	1 (1.0%)	2 (3.7%)
Other	22 (1.1%)	18 (1.2%)	3 (1.1%)	0 (0.0%)	1 (1.9%)
Average (SD) percent of people with college or more education in census block	45.1% (19.9%)	45.8% (19.7%)	43.9% (19.5%)	40.9% (21.9%)	40.8% (21.7%)
Comorbidity score					
0	1110 (57.5%)	910 (60.8%)	128 (46.2%)	54 (52.4%)	18 (33.3%)
1‐2	533 (27.6%)	400 (26.7%)	88 (31.8%)	33 (32.0%)	12 (22.2%)
3+	288 (14.9%)	187 (12.5%)	61 (22.0%)	16 (15.5%)	24 (44.4%)
First course therapy					
Surgery	1887 (97.7%)	1495 (99.9%)	277 (100.0%)	100 (97.1%)	15 (27.8%)
Radiation	24 (1.2%)	2 (0.1%)	3 (1.1%)	4 (3.9%)	15 (27.8%)
Chemotherapy	16 (0.8%)	0 (0.0%)	1 (0.4%)	2 (1.9%)	13 (24.1%)
Biologic response modulators	67 (3.5%)	7 (0.5%)	9 (3.3%)	35 (34.0%)	16 (29.6%)

**Figure 1:**
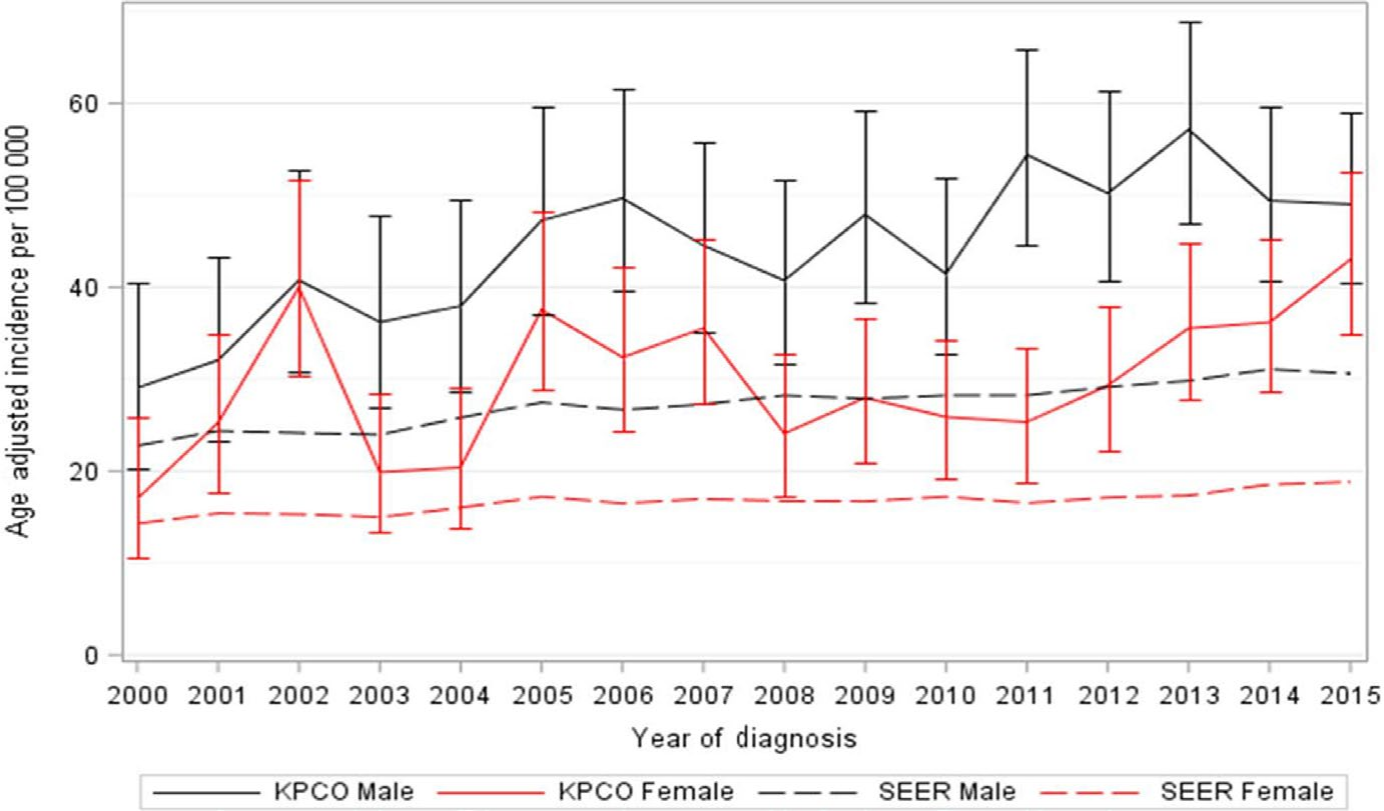
Age‐adjusted incidence rates and 95% confidence intervals (CI) for invasive melanoma, 2000‐2015. Incidence rates are displayed for the adult Kaiser Permanente Colorado (KPCO) population aged 21 years and older. SEER rates are plotted for comparison

**Figure 2:**
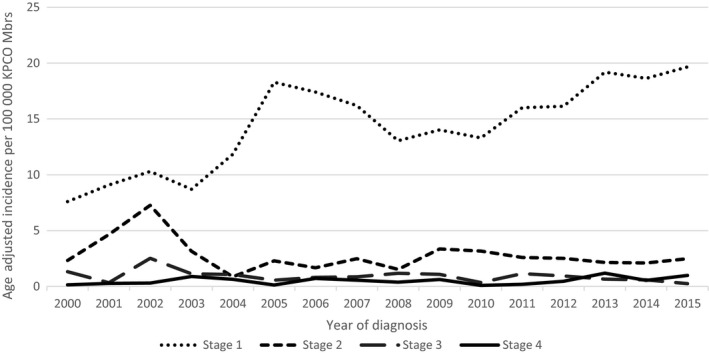
Age‐adjusted incidence rates for invasive melanoma by stage at diagnosis. Kaiser Permanente Colorado, 2000‐2015

Figure 1 shows the age‐adjusted incidence rates between 2000 and 2015 for our KPCO population by gender, and for comparison, the SEER incidence rates for the same period. The rates for KPCO are higher than SEER rates; like the SEER rates, they are increasing over time, but at a faster rate. Among men, the SEER rates increased from 22.8/100 000 in 2000 to 30.6/100 000 in 2015, compared to 29.1/100 000 to 49.0/100 000 for KPCO men in the same period. Similarly, for women, the SEER rates increased from 14.3/100 000 in 2000 to 18.8/100 000 in 2015 compared to 17.0/100 000 to 43.0/100 000 in KPCO. We observed a sharp increase in 2002 followed by a drop in 2003 that we cannot explain, otherwise, the upward trend is consistent across the entire period. Figure 2 shows age‐adjusted incidence for melanoma stratified by stage at diagnosis, and illustrates that the increased incidence over time is limited to early stage disease. Age‐adjusted incidence for stage I increased from 7.6/100 000 to 19.7/100 000 from 2000 to 2015, while the rates for stage II‐IV remained relatively constant over time.

Table 2 shows unadjusted and adjusted hazard ratios (HRs) and 95% confidence intervals (CIs) for factors associated with recurrence. Analysis of factors associated with recurrence was limited to cases diagnosed at stages I‐III. The overall rate of recurrence was 8.8%. The most commonly reported recurrence types were distant recurrence in multiple sites (20%) and local recurrences (18%) (data not shown). In multivariable models, factors associated with recurrent disease were stage at initial diagnosis, gender, and age. Race/ethnicity, socioeconomic status, and comorbidities were not associated with recurrence. In the multivariable model that did not include treatment variables, the adjusted HR was 5.54 (95% CI: 3.83‐8.03, *P* < 0.001) for stage II compared to stage I, and the adjusted HR was 18.58 (95% CI: 12.51‐27.61, *P* < 0.001) for stage III compared to stage I. Men were more likely to have a recurrence than women (adjusted HR: 1.72, 95% CI: 1.21‐2.45, *P* = 0.003), and for each decade of increasing age, the adjusted HR was 1.18 (95% CI: 1.04‐1.33, *P* = 0.009). Adding treatment to the model had little impact on the effect estimates for the other covariates. Receipt of radiation therapy was the only treatment modality associated with risk of recurrence (HR = 3.03, 95% CI: 1.10‐8.36, *P* = 0.03), while stage, male gender, and age remained as important predictors of recurrence. This increased risk associated with radiation therapy likely reflects practice patterns, as radiation is generally reserved for patients with advanced disease and for patients with large tumors who are poor surgical candidates.

**Table 2:** Hazard ratios (HR) and 95% confidence intervals (CI) for characteristics associated with recurrence for incident melanoma, stages I‐III, Kaiser Permanente Colorado, 2000‐2015

	Observations	Unadjusted HR	95% CI	Adjusted^a^ HR	95% CI	*P*‐value	Adjusted^b^ HR	95% CI	*P*‐value
Stage I	1486	Ref		Ref			Ref		
Stage II	272	6.37	4.43‐9.18	5.54	3.83‐8.03	<0.0001	5.27	3.63‐7.67	<0.0001
Stage III	94	19.54	13.24‐28.82	18.58	12.51‐27.61	<0.0001	16.23	10.45‐25.19	<0.0001
Female	788	Ref		Ref			Ref		
Male	1064	2.20	1.55‐3.12	1.72	1.21‐2.45	0.003	1.70	1.19‐2.43	0.003
Age in decades		1.29	1.15‐1.43	1.18	1.04‐1.33	0.009	1.20	1.06‐1.37	0.005
White	1816	Ref		Ref			Ref		
Non‐White	36	1.22	0.45‐3.29	1.81	0.66‐4.97	0.251	1.86	0.68‐5.12	0.228
Socioeconomic status		0.44	0.20‐0.96	0.74	0.33‐1.67	0.466	0.75	0.33‐1.69	0.486
Comorbid conditions: 0	1084	Ref		Ref			Ref		
Comorbid conditions: 1‐2	511	1.48	1.04‐2.10	1.10	0.77‐1.58	0.602	1.14	0.79‐1.64	0.494
Comorbid conditions: 3+	257	2.04	1.35‐3.08	1.12	0.72‐1.76	0.614	1.10	0.71‐1.73	0.665
No radiation	1845	Ref					Ref		
Radiation	7	10.69	3.96‐28.89				3.03	1.10‐8.36	0.0328
No chemotherapy	1849	Ref					Ref		
Chemotherapy	3	3.85	0.54‐27.62				0.80	0.11‐6.13	0.833
No biologic response modulators	1803	Ref					Ref		
Biologic response modulators	49	6.60	4.13‐10.54				1.51	0.86‐2.68	0.155

a Adjusted for stage, gender, age as a continuous variable, race/ethnicity, socioeconomic status as measured by percent of college educated households in census tract of residence, and comorbidity index

b This model adds dichotomous variables for the type of treatments received in addition to the covariates listed in the prior model. Surgery was not included as all but one case received surgery as part of treatment

Table 3 shows adjusted HRs and 95% CIs for factors associated with all‐cause and melanoma‐specific mortality. There were 342 deaths among the 1931 cohort members; the median follow‐up time was 4.1 years. In multivariable models, stage, gender, age at diagnosis, socioeconomic status as measured by education at the census tract level, and comorbidity index were all statistically significantly associated with all‐cause mortality. Risk of death increased at each stage of diagnosis; compared to stage I, the HR was 12.87 (95% CI: 6.63‐24.99, *P* < 0.001) for stage IV disease. Men were 42% more likely to die than women (HR = 1.42, 95% CI: 1.12‐1.79, *P* = 0.003). For each decade of increasing age, the HR was 1.89 (95% CI: 1.70‐2.10, *P* < 0.001), and higher socioeconomic status was protective (HR = 0.40, 95% CI: 0.23‐0.72, *P* = 0.002). The presence of three or more comorbid conditions doubled the risk of death from any cause (HR = 2.10, 95% CI: 1.57‐2.79, *P* < 0.001). Surgery and radiation both reduced the risk of death, but we did not observe an effect from chemotherapy or biologic response modulators. Factors associated with melanoma‐specific mortality were similar to those associated with all‐cause mortality. The association with disease stage was very strong for melanoma‐specific death and the presence of comorbid conditions was less important. Interestingly, age was not a significant predictor of melanoma‐specific mortality in multivariate models (HR = 1.09, 95% CI: 0.94‐1.25, *P* = 0.253).

**Table 3:** Hazard ratios (HR) and 95% confidence intervals (CI) for characteristics associated with all‐cause mortality and melanoma‐specific mortality, Kaiser Permanente Colorado, 2000‐2015

	Observations	All‐Cause HR^a^	95% CI	*P*‐value	Melanoma HR^a^^,^ b	95% CI	*P*‐value
Stage I	1497	Ref				Ref	
Stage II	277	1.79	1.37‐2.35	<0.0001	7.61	4.55‐12.74	<0.0001
Stage III	103	3.83	2.72‐5.40	<0.0001	22.50	12.89‐39.26	<0.0001
Stage IV	54	12.87	6.63‐24.99	<0.0001	98.05	40.83‐235.43	<0.0001
Female	815	Ref				Ref	
Male	1116	1.42	1.12‐1.79	0.003	1.95	1.29‐2.95	0.002
Age in decades		1.89	1.70‐2.10	<0.0001	1.09	0.94‐1.25	0.253
White	1888	Ref				Ref	
Non‐White	43	1.79	0.90‐3.55	0.095	2.66	1.01‐7.00	0.048
Socioeconomic status		0.40	0.23‐0.72	0.002	0.73	0.28‐1.90	0.516
Comorbid conditions: 0	1110	Ref				Ref	
Comorbid conditions: 1‐2	533	1.68	1.29‐2.18	0.0001	1.21	0.79‐1.86	0.370
Comorbid conditions: 3+	288	2.10	1.57‐2.79	<0.0001	1.64	1.05‐2.56	0.030
No surgery	44	Ref				Ref	
surgery	1887	0.20	0.10‐0.40	<0.0001	0.31	0.13‐0.74	0.008
No radiation	1907	Ref				Ref	
radiation	24	0.42	0.21‐0.83	0.013	0.37	0.17‐0.79	0.010
No chemotherapy	1915	Ref				Ref	
chemotherapy	16	1.03	0.50‐2.10	0.947	0.89	0.41‐1.93	0.765
No biologic response modulators	1864	Ref				Ref	
Biologic response modulators	67	1.35	0.82‐2.23	0.241	0.76	0.42‐1.38	0.369

a Adjusted for stage, gender, age as a continuous variable, race/ethnicity, socioeconomic status as measured by percent of college educated households in census tract of residence, comorbidity index, and dichotomous variables for the type of treatments received.

b Melanoma‐specific mortality model excludes 18 cases where cause of death was unknown.

In sensitivity analyses replacing tumor thickness for stage in our models of total mortality and recurrence, our results were similar (Tables S1 and S2); risk increased with thicker lesions, and the characteristics associated with recurrences and mortality remained the same with two exceptions: radiation therapy was no longer a significant predictor of recurrence, and biologic response modulators became a statistically significant predictor of recurrence.

## 4 DISCUSSION

This retrospective analysis of melanoma patients was designed to examine trends in incidence and predictors of both recurrence and survival in a large, insured population. Consistent with previous studies,^31^, ^32^, ^33^ we observed a significant increase in the incidence of melanoma between 2000 and 2015, which was largely due to increasing rates of early stage disease. KPCO incidence was higher than the reported SEER rates, and this likely reflects both our high elevation, which increases UV exposure, and better access to screening for our insured population. We did observe a sharp increase in incidence in 2002 that we do not fully understand. We did not uncover any changes in our population, clinical practices or tumor registry coding or procedures that could explain this observation; fortunately, it did not drive any conclusions. It is possible that this observation is due to chance, given the relatively small number of cases diagnosed each year.

Singh et al^34^ used data from CDC and SEER and county‐level summary socioeconomic measures from the US Census and reported that incidence was associated with lower poverty, higher education, and higher income. While we did not examine incidence rates by socioeconomic status, these observations are consistent with our data from a predominantly white, insured population. In multivariate models, we found higher socioeconomic status measured by education level in the census tract of residence was significantly associated reduced risk of death (HR: 0.40, 95% CI: 0.22‐0.72). Studies outside of the United States have had inconsistent associations with socioeconomic status and survival.^7^, ^14^

In addition to socioeconomic status, stage at diagnosis, male gender, increasing age, and the presence of comorbidities were all independent predictors of all‐cause mortality. Increased risk of death associated with increasing age and male gender have been shown consistently in prior studies.^11^, ^13^ We also found surgery and radiation therapy were associated with a reduced risk of death from all causes, but chemotherapy and biologic response modulators were not. These associations may be explained by practice patterns: nearly all our patients (97.7%) received surgery; chemotherapy and biologic response modulators were used in only 4% of patients, all of whom presented with advanced disease. Predictors of melanoma‐specific death were similar to those of all‐cause mortality, with one notable exception: age was not associated with melanoma‐specific mortality after adjusting for other covariates (HR = 1.09, 95% CI: 0.94‐1.25, *P* = 0.253). Prior studies have also found men at higher risk of death from melanoma.^10^, ^13^, ^14^ While others have not reported a lack of association with age, in a study of young adults age 15‐39 years with melanoma, Gamba et al^10^ found that men were 55% more likely to die from melanoma than women. This higher risk of death was observed across all tumor thicknesses and age ranges even after multivariable adjustment.

Several characteristics that predict mortality also predict recurrence: higher stage, older age, and male gender were all statistically significantly associated with recurrence in multivariable models, even after accounting for treatment. Men had a higher risk of recurrence compared to women (HR: 1.70, 95% CI: 1.19‐2.43), suggesting that men may benefit from more intensive follow‐up with more frequent screening intervals than women. Socioeconomic status, comorbidity, and race/ethnicity were not predictors of recurrence. Studies of melanoma recurrence are limited, and no population‐based studies have been conducted in the United States.^18^, ^19^, ^20^, ^21^, ^22^, ^23^ Findings from previous studies were consistent with ours; increasing age, male gender, and tumor thickness were important predictors of recurrence. Prior studies did not address comorbidities, race/ethnicity, nor socioeconomic status.

We observed a lower rate of recurrence in our population compared to prior studies (8.8% vs 12%‐30%).^18^, ^19^, ^23^ There are several possible explanations for differences in rate of recurrence, including differences between the study populations in distribution of stage at diagnosis, age and gender, or clinical characteristics including treatment and surveillance. The difference could also be due to differences in the definition of recurrence. Our definition is used by certified tumor registrars to monitor disease, and requires a “disease free” interval to precede a documented recurrence. Berger et al^23^ limited their analysis to cases diagnosed at stage II, and Rockberg et al^19^ included instances of disease progression in their definition of recurrence as well as cases diagnosed at stage IV. Our study is most similar to that of Lyth et al^18^ who examined prognostic factors for cases diagnosed at stages I‐II, reported a recurrence rate of 12%, and found that tumor thickness was the predominant risk factor for recurrence. Similarly, we found that stage (and in sensitivity analysis, tumor thickness) was a strong predictor of recurrence, along with gender and age.

We acknowledge the limitations of our study. We relied upon NAACCR definitions in the tumor registry to distinguish between chemotherapy and biological response modifiers, and during the study period, classifications and registry capture of some agents may have changed. Because use of any chemotherapy or biological response modifiers was uncommon in our population (~4% overall), we do not believe this biased our analysis. We also lack information on genetic susceptibility and did not examine specific mutations nor histopathologic features that have been previously associated with survival.^12^, ^15^ We did not have individual level data on education or income; thus, to asses socioeconomic status, we relied on census block level information based on home address, which may have introduced some misclassification. We have a limited number of non‐white patients in our population, and therefore, we were unable to examine differences that may exist in survival or recurrence by race/ethnicity. Finally, while our well‐defined population is a strength in many ways, it also may limit the generalizability of our findings.

Our study also has several strengths. Using our extensive data systems allows complete capture of cancer data and the ability to follow patients over an extended period of time. Our recurrence data, which are captured by tumor registrars using systematic medical record review, are a notable strength of this study. Few studies can examine recurrence rates, and thus, this adds a unique and valuable contribution to our knowledge of melanoma. Our findings reflect usual care in a community setting for an insured population. As such, access to care is unlikely to be an important confounder to our findings, and our observations may reflect a more accurate picture of disease characteristics, recurrence and survival than studies in a tertiary care setting.

To our knowledge, this is the first population‐based study in the United States to examine patient characteristics associated with risk of recurrence. We found that stage, male gender and age are associated with recurrence. These characteristics are also associated with overall survival, along with socioeconomic status and the presence of multiple comorbidities. Because men have an increased risk of both recurrence and death, they may benefit from more intensive follow‐up than women.

## CONFLICT OF INTEREST

The authors have no conflicts of interest.

## DATA AVAILABILITY STATEMENT

The data that support the findings of this study are available from the corresponding author upon reasonable request.
