# Supplementary material for: Melanoma incidence, recurrence, and mortality in an integrated healthcare system: A retrospective cohort study
Source: Cancer Med. 2019 Jun 19;8(9):4508–16. doi: 10.1002/cam4.2252 (PMC6675720; doi:10.1002/cam4.2252)
Supplement: Supplementary file 1 [file CAM4-8-4508-s001.docx]

Supplemental Table S1. sensitivity. Hazard ratios (HR) and 95% confidence intervals for characteristics associated with

recurrence for incident melanoma, stages I-III, Kaiser Permanente Colorado, 2000-2015

|  | Observations | Unadjusted HR | 95% CI | Adjusted HR^1^ | 95% CI | p-value |
| --- | --- | --- | --- | --- | --- | --- |
| 0.01 - 1.0 mm | 1303 | Ref | | Ref | |  |
| 1.01 - 2.0 mm | 305 | 2.37 | 1.49-3.79 | 2.20 | 1.38-3.52 | 0.0010 |
| 2.01 - 4.0 mm | 147 | 9.80 | 6.55-14.68 | 7.64 | 5.01-11.65 | <.0001 |
| 4.01 mm + | 86 | 20.21 | 13.30-30.72 | 14.28 | 9.11-22.38 | <.0001 |
|  |  |  |  |  |  |  |
| Female | 783 | Ref | | Ref | |  |
| Male | 1058 | 2.18 | 1.54-3.09 | 1.63 | 1.15-2.33 | 0.007 |
|  |  |  |  |  |  |  |
| Age in decades |  | 1.29 | 1.16-1.44 | 1.15 | 1.02-1.31 | 0.029 |
|  |  |  |  |  |  |  |
| White | 1805 | Ref | | Ref | |  |
| Non-White | 36 | 1.22 | 0.45-3.29 | 1.08 | 0.39-2.98 | 0.877 |
|  |  |  |  |  |  |  |
| Socioeconomic status |  | 0.44 | 0.20-0.98 | 0.61 | 0.28-1.36 | 0.227 |
|  |  |  |  |  |  |  |
| Comorbid conditions: 0 | 1076 | Ref | | Ref | |  |
| Comorbid conditions: 1-2 | 510 | 1.49 | 1.05-2.12 | 1.20 | 0.83-1.74 | 0.323 |
| Comorbid conditions: 3+ | 255 | 2.07 | 1.37-3.12 | 1.07 | 0.68-1.69 | 0.768 |
|  |  |  |  |  |  |  |
| No Radiation | 1834 | Ref | | Ref | |  |
| Radiation | 7 | 10.70 | 3.96-28.90 | 1.22 | 0.43-3.48 | 0.704 |
|  |  |  |  |  |  |  |
| No Chemotherapy | 1838 | Ref | | Ref | |  |
| Chemotherapy | 3 | 3.84 | 0.54-27.50 | 3.84 | 0.52-28.42 | 0.187 |
|  |  |  |  |  |  |  |
| No Biologic Response Modulators | 1794 | Ref | | Ref | |  |
| Biologic Response Modulators | 47 | 6.38 | 3.95-10.30 | 2.38 | 1.38-4.10 | 0.002 |

^1^Adjusted for stage, gender, age as a continuous variable, race/ethnicity, socioeconomic status as measured by percent of

college educated households in census tract of residence, comorbidity index, receipt of radiation, chemotherapy and

biologic response modulators

Supplemental Table S2. Hazard ratios (HR) and 95% confidence intervals for characteristics associated with mortality after incident

melanoma, Kaiser Permanente Colorado, 2000-2015.

|  | Observations | Unadjusted HR | 95% CI | Adjusted HR^1^ | 95% CI | p-value |
| --- | --- | --- | --- | --- | --- | --- |
| 0.01 - 1.0 mm | 1316 | Ref | Ref |  |  |  |
| 1.01 - 2.0 mm | 312 | 1.60 | 1.18-2.17 | 1.47 | 1.08-1.99 | 0.015 |
| 2.01 - 4.0 mm | 151 | 3.15 | 2.28-4.37 | 1.90 | 1.36-2.67 | 0.0002 |
| 4.01 mm + | 99 | 6.24 | 4.46-8.73 | 4.31 | 3.03-6.13 | <.0001 |
|  |  |  |  |  |  |  |
| Female | 794 | Ref | Ref |  |  |  |
| Male | 1084 | 1.95 | 1.53-2.49 | 1.48 | 1.16-1.89 | 0.002 |
|  |  |  |  |  |  |  |
| Age in decades |  | 2.18 | 1.97-2.42 | 2.06 | 1.84-2.31 | <.0001 |
|  |  |  |  |  |  |  |
| White | 1835 | Ref | Ref |  |  |  |
| Non-White | 43 | 1.48 | 0.76-2.86 | 1.79 | 0.90-3.56 | 0.097 |
|  |  |  |  |  |  |  |
| Socioeconomic status |  | 0.24 | 0.13-0.43 | 0.37 | 0.20-0.67 | 0.001 |
|  |  |  |  |  |  |  |
| Comorbid conditions: 0 | 1089 | Ref | Ref |  |  |  |
| Comorbid conditions: 1-2 | 523 | 2.41 | 1.85-3.15 | 1.48 | 1.13-1.95 | 0.005 |
| Comorbid conditions: 3+ | 266 | 4.83 | 3.64-6.40 | 1.75 | 1.29-2.38 | 0.0003 |
|  |  |  |  |  |  |  |
| No Surgery | 4 | Ref | Ref |  |  |  |
| Surgery | 1874 | 0.01 | 0.00-0.02 | 0.01 | 0.00-0.03 | <.0001 |
|  |  |  |  |  |  |  |
| No Radiation | 1870 | Ref | Ref |  |  |  |
| Radiation | 8 | 2.83 | 0.91-8.82 | 0.42 | 0.13-1.35 | 0.145 |
|  |  |  |  |  |  |  |
| No Chemotherapy | 1870 | Ref | Ref |  |  |  |
| Chemotherapy | 8 | 3.68 | 1.37-9.88 | 2.02 | 0.51-7.93 | 0.316 |
|  |  |  |  |  |  |  |
| No Biologic Response Modulators | 1825 | Ref | Ref |  |  |  |
| Biologic Response Modulators | 53 | 2.77 | 1.70-4.53 | 3.40 | 1.97-5.87 | <.0001 |

^1^Adjusted for stage, gender, age as a continuous variable, race/ethnicity, socioeconomic status as measured by percent of

college educated households in census tract of residence, comorbidity index, receipt of surgery, radiation, chemotherapy and

biologic response modulators
